# The impact of artificial intelligence on clinical education: perceptions of postgraduate trainee doctors in London (UK) and recommendations for trainers

**DOI:** 10.1186/s12909-021-02870-x

**Published:** 2021-08-14

**Authors:** Maya Banerjee, Daphne Chiew, Keval T. Patel, Ieuan Johns, Digby Chappell, Nick Linton, Graham D. Cole, Darrel P. Francis, Jo Szram, Jack Ross, Sameer Zaman

**Affiliations:** 1 University College London, Gower Street, London, WC1E 6BT UK; 2 Imperial College London, Exhibition Road, London, SW7 2AZ UK; 3 Guy’s & St. Thomas’ NHS Foundation Trust, Westminster Bridge Road, London, SE1 7EH UK; 4 Imperial College Healthcare NHS Trust, Du Cane Road, London, W12 0HS UK; 5 Royal College of Physicians, 11 St. Andrews Place, London, NW1 4LE UK; 6 Artificial Intelligence for Healthcare Centre for Doctoral Training, Imperial College London, South Kensington Campus, London, SW7 2BX UK

**Keywords:** Artificial intelligence, Machine learning, Medical education, Clinical training

## Abstract

**Background:**

Artificial intelligence (AI) technologies are increasingly used in clinical practice. Although there is robust evidence that AI innovations can improve patient care, reduce clinicians’ workload and increase efficiency, their impact on medical training and education remains unclear.

**Methods:**

A survey of trainee doctors’ perceived impact of AI technologies on clinical training and education was conducted at UK NHS postgraduate centers in London between October and December 2020. Impact assessment mirrored domains in training curricula such as ‘clinical judgement’, ‘practical skills’ and ‘research and quality improvement skills’. Significance between Likert-type data was analysed using Fisher’s exact test. Response variations between clinical specialities were analysed using k-modes clustering. Free-text responses were analysed by thematic analysis.

**Results:**

Two hundred ten doctors responded to the survey (response rate 72%). The majority (58%) perceived an overall positive impact of AI technologies on their training and education. Respondents agreed that AI would reduce clinical workload (62%) and improve research and audit training (68%). Trainees were skeptical that it would improve clinical judgement (46% agree, *p* = 0.12) and practical skills training (32% agree, *p* < 0.01). The majority reported insufficient AI training in their current curricula (92%), and supported having more formal AI training (81%).

**Conclusions:**

Trainee doctors have an overall positive perception of AI technologies’ impact on clinical training. There is optimism that it will improve ‘research and quality improvement’ skills and facilitate ‘curriculum mapping’. There is skepticism that it may reduce educational opportunities to develop ‘clinical judgement’ and ‘practical skills’. Medical educators should be mindful that these domains are protected as AI develops. We recommend that ‘Applied AI’ topics are formalized in curricula and digital technologies leveraged to deliver clinical education.

**Supplementary Information:**

The online version contains supplementary material available at 10.1186/s12909-021-02870-x.

## Background

The healthcare sector is undergoing a digital revolution [1], with a wide range of artificial intelligence (AI) innovations increasingly encountered in routine clinical practice [2]. From designing and training algorithms, to interpreting their output in clinical practice, these technologies require skillful human-machine interaction. The need for formal AI training has been recognized as a priority for government health policy [3, 4]. Although there is an abundance of evidence supporting the safety, accuracy, cost and workflow benefits of AI technologies in healthcare [5–7], there is limited research investigating the impact of AI on medical education [8, 9], the new skills required, and in particular the perceptions of trainee doctors who will be frontline users of AI in healthcare.

Increasingly, AI technologies are being developed that are capable of automating many tasks typically performed by doctors as part of their clinical training. So far these advancements are mostly limited to research applications, but it is only a matter of time before commercial uptake results in their routine use in everyday clinical practice. For example, decision support systems can triage patients, suggest diagnoses and alert test results by analysing data faster and more accurately than clinicians [6, 10]. Natural language processing can automate clinical documentation by text summarization [11] and detect diagnoses from scan reports [12]. Computer vision algorithms can detect lesions from radiological scans, make measurements, and pick-up incidental findings, saving time and reducing error [13, 14]. Robots trained by human operators could perform procedures such as venepuncture, and ultrasound [15, 16].

Although these technologies undoubtedly offer considerable benefits to patients, clinicians, and healthcare systems, there are concerns that they could come at a cost such as lack of trust in the algorithmic output due to the black-box effect [17, 18]. AI could also *reduce* training opportunities for doctors to develop clinical judgement, practical ability and communication skills. Current training programmes may fall short of equipping clinicians with the technical, statistical and analytical skills required to apply AI effectively for themselves and their patients.

The length of postgraduate medical training varies between countries, but the content of medical curricula is largely consistent globally [8]. Traditionally, medical education is centred around knowledge assimilation (by didactic teaching) and vocational training to develop practical, interpersonal and professional skills [19]. With AI algorithms capable of retaining and computing many orders of magnitude more data than humans, there may be less need for clinicians to memorize large amounts of medical information or to hone procedural skills by repetitive practice. Conversely, additional skills are required to interact safely and effectively with AI technologies such as data science, statistics and AI ethics [8]. Tomorrow’s clinicians must be skilled in data input, interpreting algorithmic outputs and communication of AI-derived treatment plans to patients [20]. Although formal education in these skills is recommended in national healthcare policy [2], it does not currently feature in most clinical training curricula.

In this study we evaluate the impact of AI technologies on clinical education, as perceived by doctors in postgraduate training. Based on the results, we make practical recommendations for trainers to maximise the benefits of clinical AI whilst mitigating potential negative impacts to deliver future-proof medical education.

## Methods

### Survey

A questionnaire was devised to investigate trainee doctors’ perceptions of AI technologies, specifically the impact on their training and education. The survey was sent by email to doctors working in London (UK) at NHS postgraduate training centers at which research team members were based to maximise response rate. These centers, mainly headquartered at tertiary hospitals, administrate both hospital and community-based postgraduate training in London, enabling inclusion of trainees from a wide range of specialities. The survey was conducted between October and December 2020. Respondents provided their informed consent by participating in the anonymous survey. The rationale, protocol and data collection tool were approved by the Guy’s and St. Thomas’ NHS Foundation Trust Institutional Audit and Quality Improvement Review Board (ref. 11,865). All methods were carried out in accordance with relevant guidelines and regulations.

Demographic data such as age, sex, clinical specialism and geographical location were anonymously collected. Five-point Likert-type scales (ranging from ‘strongly disagree’ to ‘strongly agree’) were used to investigate participants’ agreement with statements across a number of domains. The content validity of Likert-type scale statements was assessed by an expert panel of six healthcare professionals with experience of AI in healthcare. Individual items were scored to reach a consensus on which should be included. The survey questions were designed to minimize bias and further refined based on a pilot study of participants. The domains of impact assessment mirrored common themes in UK clinical training curricula: ‘clinical judgement’, ‘practical skills’, ‘research and quality improvement skills’ and ‘curriculum mapping’. Non-mandatory free-text spaces provided respondents the opportunity to comment on the positive and negative impacts of AI on clinical training.

### Quantitative analyses

Internal reliability of the survey questions was measured by calculating Cronbach’s alpha. Likert-type data were analysed by (i) calculating the category of the median response for each statement, and (ii) comparing the proportion of agreement between the overall perception statement (‘AI systems being used in healthcare will improve my training and education’) with the other statements in a pairwise manner using Fisher’s exact test. To display the range of responses across each question, waffle plots were generated using the matplotlib library in Python 3.8. K-modes clustering was used to identify key groups of responses to the Likert-type statements [21].

### Qualitative analyses

Free-text responses were analysed using thematic analysis [22]. Text data were hand-coded by two independent researchers. Themes and sub-themes were subsequently generated and representative examples identified from the raw data.

## Results

### Quantitative analyses

**Fig. 1:**
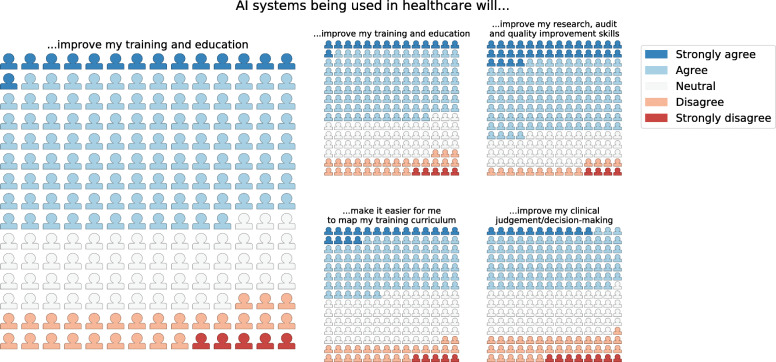
Domain-based impact of clinical AI on training and education - waffle plots of responses to Likert-type questions in survey of 210 trainee doctors (each icon represents one respondent to the survey)

**Fig. 2:**
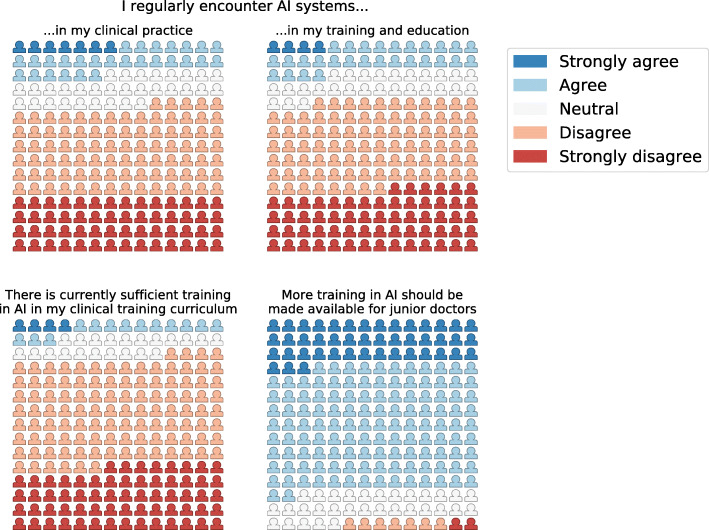
Exposure to AI systems and attitudes towards AI training - waffle plots of responses to Likert-type questions in survey of 210 trainee doctors (each icon represents one respondent to the survey)

Two hundred ten doctors responded to the online survey (response rate 72%; 47% female). Overall there was high internal reliability of the survey questions (Cronbach’s alpha 0.82). 58% perceived an overall positive impact of AI technologies on clinical training (Fig. 1). Trainees agreed that AI would reduce clinical workload (62%) and improve training in research and audit skills (68%). Lower proportions agreed that it would improve training in clinical judgement (46%) and practical skills (32%) (Fig. 1). The majority reported insufficient AI training in their current curricula (92%), and supported more formal AI training (81%) (Fig. 2). Detailed responses to all Likert-type questions in the survey are available in Supplementary Appendix A (Figures 1 and 2). The median responses to the Likert-scale questions are shown in Table 1. There was agreement that AI would have an overall positive impact on training and education. This agreement was sustained for ‘research and audit skills’ and ‘curriculum mapping’. Conversely, respondents tended towards disagreement for the domains of ‘clinical judgement/decision making’ (*p* = 0.12) and strong disagreement for the domain ‘clinical skills’ (*p* < 0.01) (Table 2).

**Table 1:** Median response of participants answering the Likert-type statements

Likert-type statement	Median response
**I regularly encounter AI systems in my:**	
Clinical practice	Disagree
Training and education	Disagree
**AI systems being used in healthcare will:**	
**Improve my training and education**	**Agree**
Reduce my clinical workload	Agree
Improve my clinical judgement/decision-making	Neutral
Improve my practical skills	Neutral
Improve my research, audit and quality improvement skills	Agree
Make it easier for me to map my training curriculum	Neutral
**Training in AI**	
There is currently sufficient training in AI in my clinical curriculum	Disagree
More training in AI should be made available	Agree

**Table 2:** Proportion of participants agreeing with statements (agree and strongly agree). *P*-values of pairwise Fisher’s exact test comparisons of % agreement between the overall statement (bold) and other statements

Likert-type statement AI systems being used in healthcare will:	Agreement (%)	***P***-value (in comparison to overall perception)
**Improve my training and education**	**58.4**	–
Reduce my clinical workload	61.8	0.67
Improve my clinical judgement/decision-making	45.8	0.12
Improve my practical skills	31.9	< 0.01
Improve my research, audit and quality improvement skills	68.1	0.19
Make it easier for me to map my training curriculum	49.2	0.26

### Cluster analyses

Clustering of the Likert-type statement responses revealed four key groups:

- AI Negative (8 participants) - strongly disagreed with the majority of the statements.
- AI Indifferent (84 participants) - no preference toward agree or disagree.
- AI Optimistic (105 participants) - a preference toward agreeing with statements.
- AI Positive (15 participants) - strongly agreed with the majority of the statements.

**Fig. 3:**
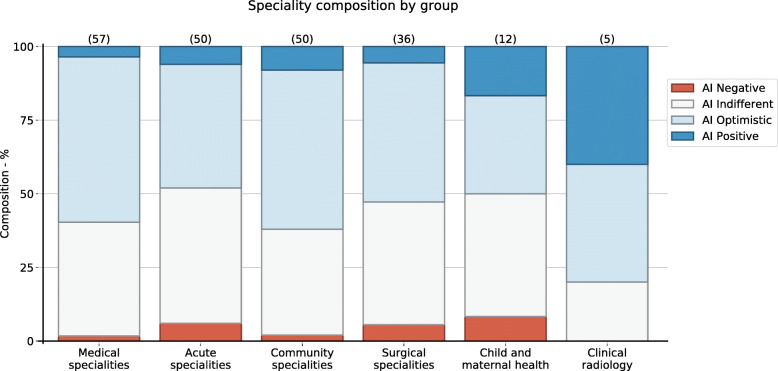
The Likert-type statement responses cluster composition of different clinical specialities. (Acute specialities include Acute Medicine, Intensive Care Medicine, Anaesthetics and Emergency Medicine; Child and maternal health include Paediatrics, Obstetrics and Gynaecology; Community specialities include General Practice and Psychiatry)

The cluster composition of clinical specialisms was calculated (Fig. 3), and shows that community-based and radiology specialisms contained a higher proportion of ‘AI Optimistic’ participants. Acute specialities and child and maternal health specialities contained a higher proportion of ‘AI Indifferent’ participants. The strongly polarized groups of ‘AI Positive’ and ‘AI Negative’ are much smaller in size in all specialisms except for Clinical Radiology in which there was a high proportion of ‘AI positive’ participants. This result should be interpreted in the context of the small proportion of respondents from this specialty.

### Qualitative analyses

**Fig. 4:**
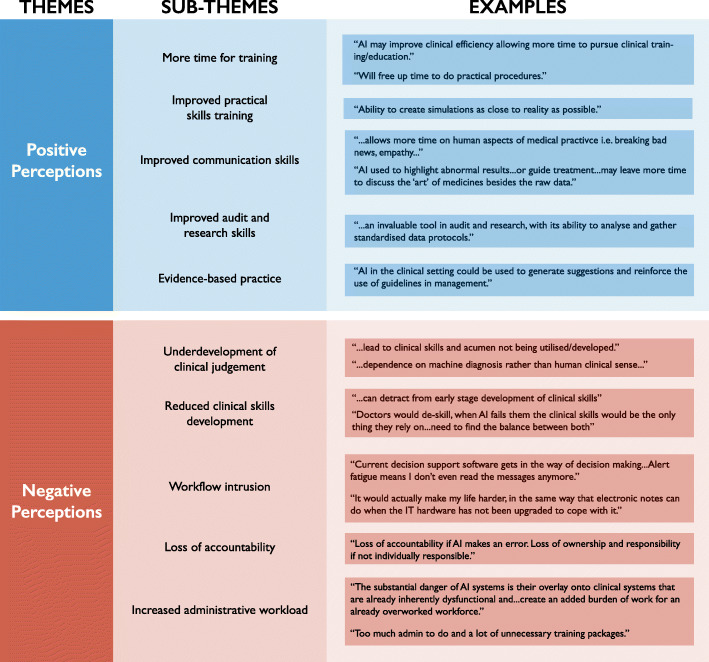
Themes and sub-themes identified from thematic analysis of free-text response data, along with representative examples from the raw data

Thematic analysis of free-text responses revealed two main themes (‘positive perceptions’ and ‘negative perceptions’) (Fig. 4). Positive sub-themes included more free time for training and educational activities, directly improving the quality of practical skills training by enabling high-fidelity simulation, enabling more time to engage in interpersonal aspects of clinical practice, and boosting training in research, audit and quality improvement.

Negative sub-themes included less development of clinical judgement, reduced opportunity for practical skills, workflow intrusion, increased administrative workload and reduced development of clinical accountability and probity.

## Discussion

This study is the first of its kind to evaluate the impact of AI technologies in healthcare on medical education and clinical training by analysing perceptions of trainee doctors across a range of hospital and community-based specialities in London, UK. Overall, doctors perceive that clinical AI will have a positive impact on their training (58% agree). Domain-based analysis reveals more mixed perceptions. The overwhelming majority (82%) report insufficient training in AI topics, with strong support for formal training in these topics.

### Domains of perceived positive impact

Doctors were most optimistic that clinical AI would improve training in research, audit and quality improvement. These are key education domains and can be challenging to fulfill without significant time and effort commitment outside of work. AI systems can improve the efficiency of research and audit by rapidly and accurately analysing large volumes of data. This may explain trainees’ positive perceptions in this regard [23]. An indirect but desirable effect is that clinical AI could free up doctors’ time to spend doing other educational activities, which was a common positive theme throughout.

Developing skills in evidence-based medicine is a key training requirement, but keeping up with rapidly changing clinical guidelines can be burdensome for doctors. AI-based decision support systems are automatically updated with the latest literature. Trainees perceived this to be a positive impact, enabling them greater exposure to the latest evidence to improve their knowledge and the quality of care they provide.

### Domains with skeptical perceptions

Trainees perceived that clinical AI could reduce their training in practical skills, clinical judgement and decision-making. Developing these skills requires iterative practice, formation of heuristics, personal reflection, varied clinical experience and time. Participants reported that decision support systems, robotics and automatic image analysis could reduce training opportunities in these domains leading to deskilling. Another skeptical perception was increased administrative workload leading to information overload. Clinical AI developers should ensure that these technologies do not impede workflow to enable their adoption in clinical practice.

Medical educators should note that there are some areas in which training may be harmed by clinical AI. Involving clinicians in the development of these algorithms (such as ground-truth labelling and procedural training) will help trainees continue to develop these skills, because the AI will depend on *their* training to mimic behavior. This will also increase trust in the AI technologies and improve explainability to patients.

### Impact on interpersonal and ethical development

The impact on interpersonal and ethical skills training featured in both positive and negative perception themes. Doctors envisage that AI will automate tasks, freeing up more time to develop communication skills. Trainees are optimistic that this will enhance their ability to provide patient-centred care. Conversely, doctors express concern that accountability is unclear when AI is part of clinical decisions, which could cause human deskilling in ownership and probity. Both points of view are valid; AI in healthcare has already created a new ethical landscape [24]. Governing bodies and medical educators should work collaboratively to produce ethical and legal frameworks that will protect and enable clinicians to develop these skills effectively in the age of clinical AI.

### Perceptual variations by clinical specialism

Medical, surgical and community-based (General Practice, Psychiatry) specialities had a higher proportion of ‘AI optimistic’ trainees Fig. 3). This may be due to a lower clinical acuity in these specialities and higher administrative workload. Trainees’ in these specialities may perceive that AI will improve their workflow to free up time for training and educational activities such as communication skills development.

Acute specialities (Emergency Medicine, Acute Medicine, Anesthesia, Intensive Care) and child and maternal health (Paediatrics, Obstetrics and Gynaecology) had more ‘AI Negative’ and ‘Indifferent’ responses. This might be due to the higher clinical acuity in these specialities, including emergency procedures and diagnostic ambiguity, which may rely on experience or ‘gut-feeling’. These skills are notoriously hard to model for AI development [25]. This mirrors trainees’ perceptions that training in practical procedures and clinical judgement might be reduced by clinical AI.

Clinical Radiology trainees, although a small proportion of respondents, were highly optimistic. This echoes positive attitudes reported previously [26]. Radiology has experienced the most AI advances in clinical practice already [27, 28] so Radiology trainees are most likely to already first-hand experience of AI’s impact on their training. This may explain their positive perceptions compared to other specialities.

Overall, specialities with higher capacity for automation were more optimistic; the implication is that rather than a panacea, delivery of medical education in the AI age will need to be tailored to these subtle variations between specialities.

### Implications for clinical curricula and recommendations for trainers

As clinical practice changes so must clinical education. This study brings the need for formal AI training in clinical curricula into sharp relief, and confirms a willing appetite for this from trainee doctors. The impact of AI on medical education can take different routes. *Direct* routes leverage AI technology to improve the delivery of training itself. *Indirect* routes benefit education by streamlining workflow to free up more time for education and training. Although the majority (72%) of respondents in our survey were yet to regularly encounter AI systems in their training and education, it is an area of active research ranging from assisted radiology teaching [29] to virtual reality for surgical skills development [30] and automated assessment of procedural performance [31]. Medical curricula should be reviewed to leverage these technologies to directly improve the delivery of clinical education.

We propose that medical curriculum makers consider a new set of AI-specific skills. These include data input and management, mathematics and statistics, communicating AI outcomes to patients and AI-specific ethics. Medical training curricula are already saturated with limited room for new topics so practical training in ‘Applied AI’ would be most feasible. Alongside an overview of common ML architectures, this should include balanced training in clinical AI interpretation including data bias, overfitting and the potential for harm.

Navigating the current ML research landscape is challenging. Common pitfalls include over-optimistic conclusions from ‘human versus clinician’ studies that are usually retrospective and prone to bias [32], lack of standardized benchmarks and no universally accepted AI evaluation metrics [33]. Training in ‘Applied AI’ must additionally equip clinicians with skills in critical literature analysis.

**Fig. 5:**
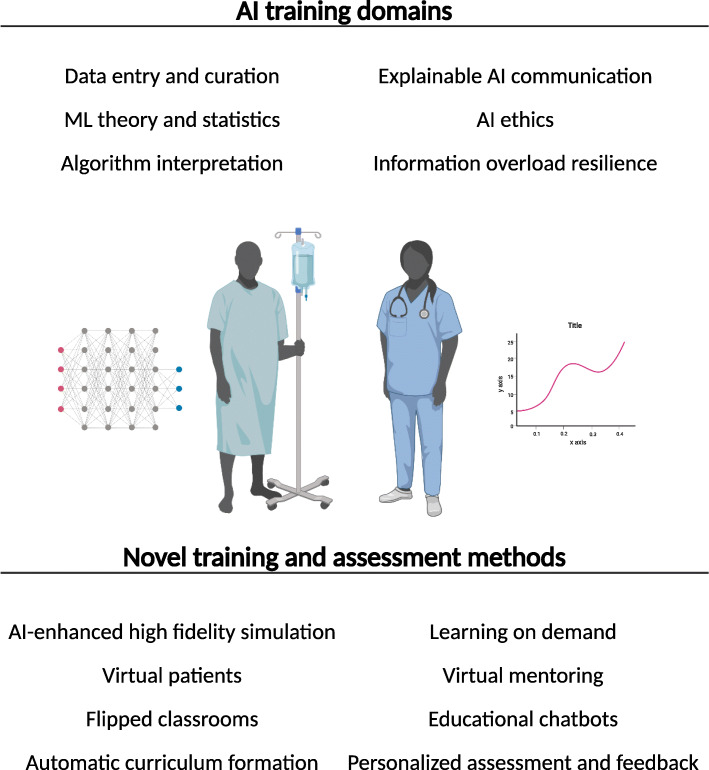
Potential new AI-related clinical training domains in future curricula. Novel training and assessment methods to deliver AI-based training. (ML = machine learning). (Created with BioRender.com)

Training in ‘Applied AI’ will need to be supported by e-learning, didactic teaching, assessment in examinations, supervised clinical learning events and personal reflection (Fig. 5). Although this has been considered as a priority for the future health workforce [2, 3], ‘Applied AI’ topics remain widely absent from clinical training curricula [9, 19]. Ultimately this will negatively affect the quality of patient care by missing out on the myriad benefits of clinical AI systems.

Our survey was not prefaced by any educational material on AI, for two reasons. First, it avoided prolonging the time participants would need to commit, which would have reduced response rates. Second, it would have biased the responses towards taking the point of view implicitly expressed in the educational material. Even if we had striven for neutrality, it would be difficult to achieve it and even more difficult to document that we had achieved it.

The optimistic perceptions of AI reported in this study may lessen as doctors gain more first-hand experience of AI in their clinical practice and training (including its weaknesses such as data bias and the black-box effect) [20]. Conversely, the opposite may occur; indeed in this study the participants working in the speciality most likely to have already experienced AI technologies (Radiology) were actually the most ‘AI optimistic’. Curriculum development must provide a balanced view, recognizing AI limitations, weaknesses and potential for harm.

### Limitations

This study provides a snapshot of the opinions of trainee doctors working in the UK NHS. The survey was hosted primarily by postgraduate training centers in London (UK), where research team members were based to maximise response rate (72%). Although these centers administrate postgraduate training for hospital *and* community based trainees (enabling representation of trainees in General Practice, Psychiatry etc.), the results of our survey could be biased in favor of trainees working in London, who may have specific experiences due to local uptake of AI technologies in urban compared to rural areas. Based on our results, we recommend a survey of all UK NHS postgraduate centers to gain a cross-sectional representation of trainee experience, and to elucidate any geographical variations in experience and opinion. We also recommend the inclusion of medical undergraduate students, since they are the clinical workforce of the future, and most likely to be directly affected by the impact of AI technologies on medical training.

Participants’ level of AI knowledge was not collected in our survey. Participants’ role at the time of responding was collected, with only 2 respondents being currently involved with clinical AI research. The majority of respondents neither encountered AI technologies regularly in their clinical practice (68%) or training (72%). Future work would benefit from participants self-rating their level of AI knowledge to contextualize findings.

The impact on communication and interpersonal skills was not assessed in the Likert-type part of our survey, Thematic analysis of free-test responses revealed important trainee perceptions in these areas. Future evaluation of trainees’ attitudes should further probe the perceived impact on domains such as communication, professionalism, leadership and probity, which are key elements of all clinical training curricula.

## Conclusions

Clinical AI will affect medical education and clinical training. Trainee doctors have overall positive perceptions of this impact. Training in practical procedures and clinical judgement may be reduced by clinical AI and educational opportunities in these skills should be protected. There is overwhelming support for formal education in AI-based skills and topics. Medical curricula should be reviewed to include ‘Applied AI’ topics, using digital technologies to deliver training.

## Supplementary Information

**Additional file 1: Appendix A.** Responses to Likert-type questions in the survey of trainee doctors.

## Data Availability

The datasets used and/or analysed during the current study are available from the corresponding author on reasonable request.

## Abbreviations

AI: Artificial intelligence
ML: Machine learning
