# Supplementary material for: The impact of artificial intelligence on clinical education: perceptions of postgraduate trainee doctors in London (UK) and recommendations for trainers
Source: BMC Med Educ. 2021 Aug 14;21:429. doi: 10.1186/s12909-021-02870-x (PMC8364021; doi:10.1186/s12909-021-02870-x)
Supplement: Supplementary file 1 — Additional file 1: Appendix A. Responses to Likert-type questions in the survey of trainee doctors. [file 12909_2021_2870_MOESM1_ESM.docx]

**Appendix A: Responses to Likert-type questions in the survey of trainee doctors**

**
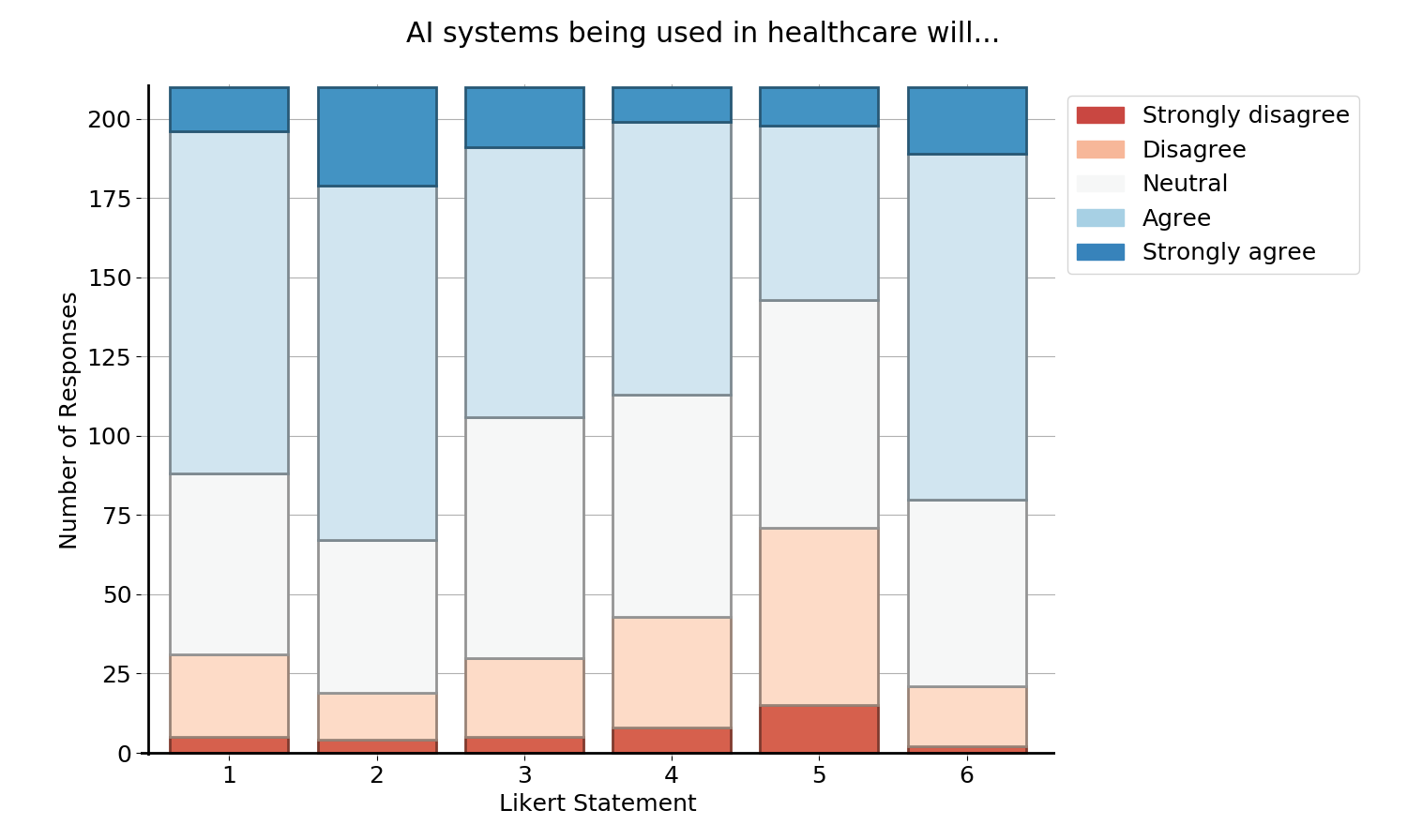
**

Figure 1: 'AI systems being used in healthcare will...'

| *Number* | *Likert Statement* |
| --- | --- |
| *1* | *… improve my training and education.* |
| *2* | *… make it easier for me to map my training curriculum.* |
| *3* | *… improve my research, audit and quality improvement skills.* |
| *4* | *…improve my clinical judgement/decision-making.* |
| *5* | *… improve my practical skills.* |
| *6* | *… will reduce my clinical workload.* |

**
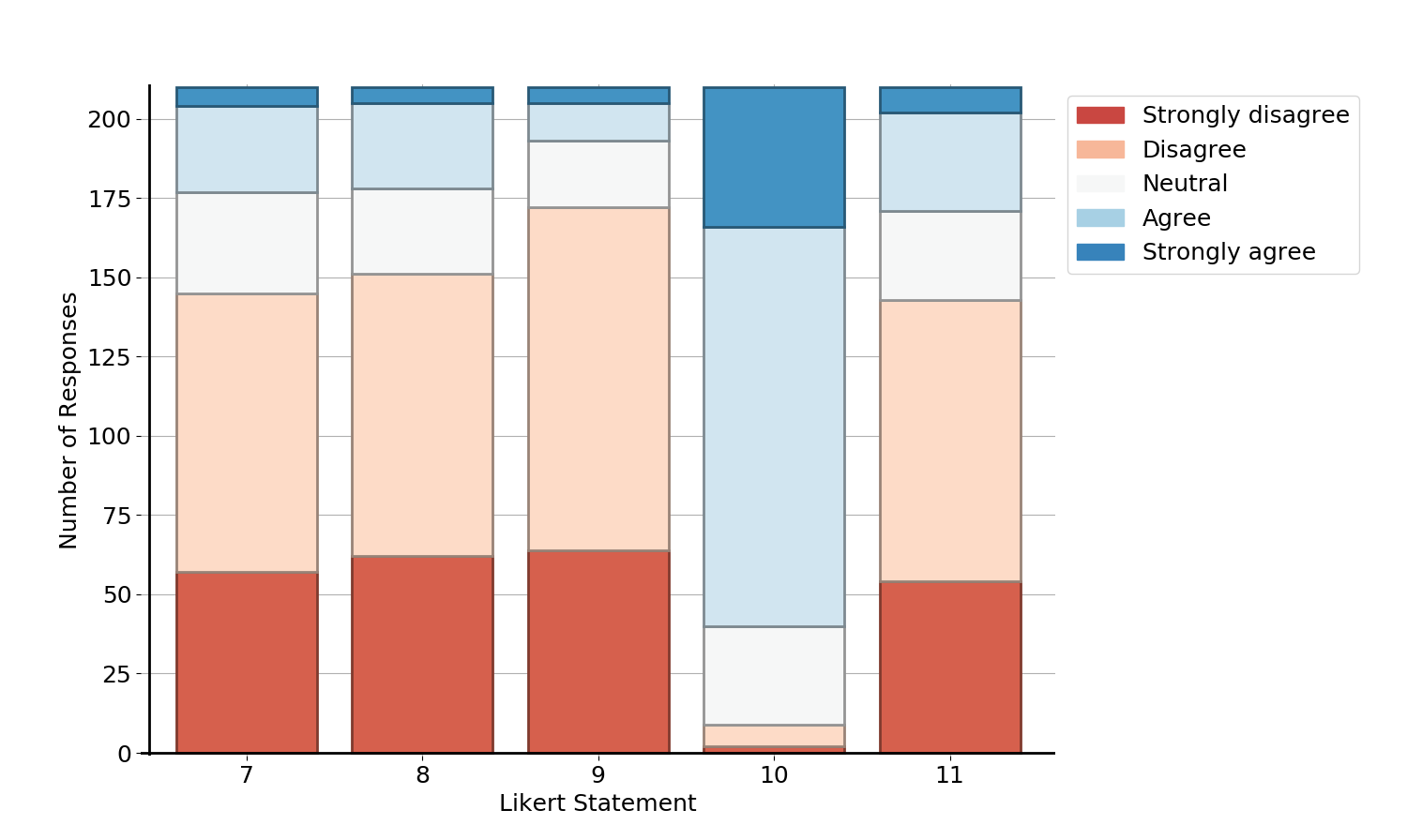
**

Figure 2:

| *Number* | *Likert Statement* |
| --- | --- |
| *7* | *I regularly encounter AI systems in my clinical practice.* |
| *8* | *I regularly encounter AI systems in my training and education.* |
| *9* | *There is currently sufficient training in AI in my clinical training curriculum.* |
| *10* | *More training in AI should be made available for junior doctors.* |
| *11* | *I know where the responsibility lies when AI systems are used in clinical practice.* |
